# Predicting the Impact of OTOF Gene Missense Variants on Auditory Neuropathy Spectrum Disorder

**DOI:** 10.3390/ijms242417240

**Published:** 2023-12-07

**Authors:** Dmitry A. Dmitriev, Boris V. Shilov, Michail M. Polunin, Anton D. Zadorozhny, Alexey A. Lagunin

**Affiliations:** 1 Department of Bioinformatics, Medico-Biological Faculty, Pirogov Russian National Research Medical University, Moscow 117997, Russia; dmitrii_d@mail.ru (D.A.D.); boris.shilov@gmail.com (B.V.S.); zadorozhnyy.ad@gmail.com (A.D.Z.); 2 Department of Otorhinolaryngology, Faculty of Pediatrics, Pirogov Russian National Research Medical University, Moscow 117997, Russia; polunin_mm@rsmu.ru; 3 Department of Bioinformatics, Institute of Biomedical Chemistry, Moscow 119121, Russia

**Keywords:** sensorineural hearing loss, auditory neuropathy spectrum disorder, OTOF, otoferlin, machine learning, missense variants, amino acid substitutions

## Abstract

Auditory neuropathy spectrum disorder (ANSD) associated with mutations of the OTOF gene is one of the common types of sensorineural hearing loss of a hereditary nature. Due to its high genetic heterogeneity, ANSD is considered one of the most difficult hearing disorders to diagnose. The dataset from 270 known annotated single amino acid substitutions (SAV) related to ANSD was created. It was used to estimate the accuracy of pathogenicity prediction using the known (from dbNSFP4.4) method and a new one. The new method (ConStruct) for the creation of the protein-centric classification model is based on the use of Random Forest for the analysis of missense variants in exons of the OTOF gene. A system of predictor variables was developed based on the modern understanding of the structure and function of the otoferlin protein and reflecting the location of changes in the tertiary structure of the protein due to mutations in the OTOF gene. The conservation values of nucleotide substitutions in genomes of 100 vertebrates and 30 primates were also used as variables. The average prediction of balanced accuracy and the AUC value calculated by the 5-fold cross-validation procedure were 0.866 and 0.903, respectively. The model shows good results for interpreting data from the targeted sequencing of the OTOF gene and can be implemented as an auxiliary tool for the diagnosis of ANSD in the early stages of ontogenesis. The created model, together with the results of the pathogenicity prediction of SAVs via other known accurate methods, were used for the evaluation of a manually created set of 1302 VUS related to ANSD. Based on the analysis of predicted results, 16 SAVs were selected as the new most probable pathogenic variants.

## 1. Introduction

Hearing impairment is currently the most common congenital sensory pathology. The World Health Organization estimates that by 2050, more than 700 million people will have disabling hearing loss (WHO, 2021, https://www.who.int/news-room/fact-sheets/detail/deafness-and-hearing-loss (accessed on 5 November 2023)). According to the results of epidemiological studies, the prevalence of congenital sensorineural hearing loss ranges from one to two cases per thousand newborns [1]. Hearing pathology has a negative impact on the process of speech acquisition and the cognitive and psycho-emotional development of the child, leading to social isolation and difficulties in adapting to the outside world. The early diagnosis of hearing disorders is crucial for organizing timely treatment and rehabilitation measures, the effectiveness of which largely depends on the plasticity of the nervous system structures characteristic in young children. Thus, a current area of research is the development and implementation of reliable diagnostic tools that make it possible to detect hearing impairment in the early stages of child development.

One of the common types of sensorineural hearing loss is Auditory Neuropathy Spectrum Disorder (ANSD), which is characterized by the registration of otoacoustic emissions (OAE) and/or cochlear microphone potential (MP) in combination with the absence or change in short-latency auditory-evoked potentials (SAEPs). Impaired speech perception is considered an important clinical feature of ANSD despite the relative preservation of pure tone detection thresholds [2]. The diagnosis of ANSD is also complicated by limitations associated with the assessment of auditory perception at the prelingual stage of child development.

ANSD is detected in 10% of cases among children suffering from sensorineural hearing loss. This indicator increases to 15–20% in children with severe hearing loss [3,4,5]. Due to its high genetic heterogeneity, ANSD is regarded as one of the most difficult hearing disorders to diagnose. The hereditary nature of ANSD can be associated with the presence of pathogenic variants in the ATP1A3, DIAPH3, OPA1, PJVK, and OTOF genes that determine the pathophysiological mechanism and clinical picture of the disease. The most common cause of ANSD is variants in the OTOF gene, which encodes the otoferlin protein [6]. The otoferlin protein is predominantly expressed in the human inner ear, where it plays an important role in the functioning of synapses between inner hair cells (IHCs) and auditory neurons.

The genetic testing of young children aimed at searching for pathogenic variants in the OTOF gene has an important diagnostic value due to the obvious limitations in the use of subjective psychoacoustic methods (for example, speech or pure-tone audiometry). At the same time, currently, existing studies of OTOF gene variants are not capable of providing a solution to the problem of the molecular genetic diagnosis of ANSD with high accuracy and reliability.

The prevalence of false-positive and false-negative diagnostic results is due, on the one hand, to the existence of mutations in alternative genes and, on the other hand, to the lack of data on pathogenic variants of the OTOF gene. Currently, there is a continuous search for new variants of the OTOF gene associated with ANSD. The known pathogenic variants in OTOF are related not only to ANSD but also to other hearing disorders. The known bioinformatic tools predict the general pathogenicity of single amino acid substitutions (SAV) and could be related to different hereditary diseases associated with hearing loss. A bioinformatics analysis of currently available data can be used to establish patterns of influence of OTOF gene variants on the development of ANSD and consequently increase the accuracy and reliability of the clinical and laboratory diagnoses of this disease.

In this study, we create a new protein-centric classification model using the Random Forest machine learning algorithm to solve the problem of predicting ANSD risk based on a system of predictor variables related to features of the structure and functions of the otoferlin protein in the auditory analyzer. The classification model was built on the basis of regularly updated public databases of gene variants associated with hereditary non-syndromic hearing loss. This study used known genetic missense variants related to OTOF that were available at the time of the study (August 2023).

## 2. Results

### 2.1. Creation, Validation, and Selection of the Best Classification Models

The set from 270 SAVs, including 93 pathogenic and 177 benign variants, was used to create and validate OTOF-specific classification models. The four popular machine learning methods (Random Forest (RF), Bayes, Support Vector Machines (SVM), and Probabilistic Neural Network (PNN)) were used for the creation of the models. The developed method employed data on the nucleotide conservativity and structural features of protein and was named ConStruct (conservativity and structure features). We also used the previously developed SAV-Pred method for the creation and validation of the OTOF-specific classification model [7,8]. SAV-Pred is based on the representation of molecular fragments (25 amino acid residues) of the protein as a structural formula describing substructural atom-centric MNA descriptors of the 10th level and the Bayesian-like algorithm [7,8]. The comparison of prediction accuracy was also made based on the result of the five-fold cross-validation (5-F CV) approach.

In order to compare the OTOF model performance and leading bioinformatic prediction tools, the records for the canonical ENST00000272371 transcript from the academic version of dbNSFP4.4 [9] were extracted. The pathogenicity scores for 270 substitutions from the dataset were obtained, and measures of precision were calculated (Table 1).

Table 1 shows that the application of RF to the creation of classification models (ConStruct, RF) provided the best accuracy in 5-F CV (BA—0.866, accuracy—0.881, AUC—0.903) among the four machine learning methods used (four top rows of Table 1). These values of accuracy are also higher than the ones calculated for 5-F CV results via SAV-Pred (BA—0.801, Accuracy—0.796, AUC—0.876) obtained from the same data. Unfortunately, it was not possible to reproduce the 5-F CV procedure for other methods, so for them, only an indirect comparison of accuracy was possible based on the data presented in dbNSFP4.4. In this case, many of the 270 known variants might be used in the training of the appropriate methods, and their values of accuracy could be overestimated. Nevertheless, the balanced accuracy value (0.866) of the created model (ConStruct, RF) lies close to the best values of other methods, i.e., MetaLR (0.876), MetaSVM (0.868), MutationAssessor (0.869), PolyPhen 2 HDIV (0.879), and PROVEAN (0.868). The high balanced accuracy value indicates that both sensitivity and specificity values are high, and the prediction of pathogenic variants does not come at the expense of the overprediction of benign variants and vice versa. Table 1 also shows that some of the known methods did not provide sufficiently high accuracy for SAVs of otoferlin when the balanced accuracy was less than 0.8 (FATHMM, 0.728; M-CAP, 0.724 and MVP, 0.679). Therefore, we cannot recommend the use of such methods to estimate the pathogenicity of otoferlin missense mutations.

**Figure 1:**
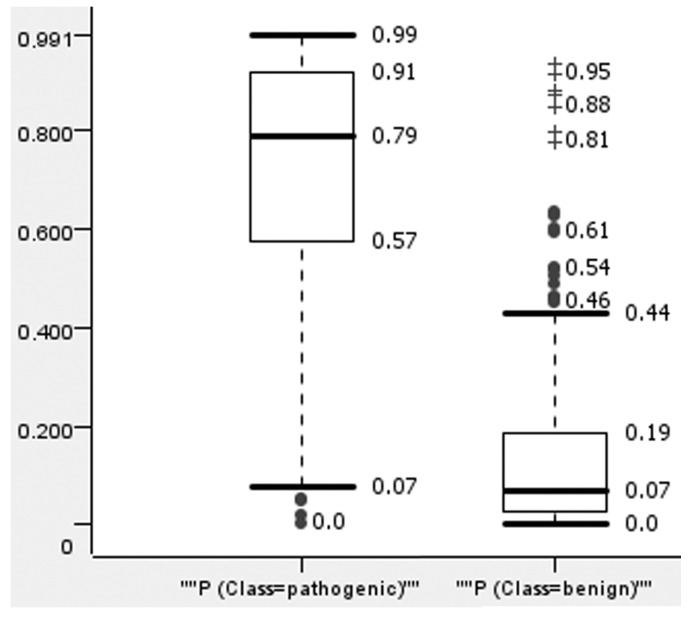
The distribution of ConStruct (RF) prediction results in 5-F CV for pathogenic and benign variants. “+” means extreme outliers; ● means mild outliers.

To reduce false positive predictions, we selected a threshold of predicted values for the created model. For this, we analyzed the distribution of the predicted values for pathogenic and benign variants in the data given for test sets in the 5-F CV procedure (Figure 1).

Figure 1 demonstrates the significant difference between the prediction values for benign and pathogenic variants. The pathogenic variants tend to be predicted with high values of probability and vice versa; benign variants were predicted mainly with low values of probability. The most pathogenic variants were predicted with a value higher than 0.57, and the most benign variants were predicted with a value less than 0.19. The extreme outliers of benign variants had values over 0.8. When selecting a threshold over 0.85, only five predictions were found to be false positives, making up a small part (12.5%) of the 40 variants predicted as pathogenic. Similar plots for other methods are represented in the Supplementary Materials (Supplements, Figures S1–S12).

### 2.2. Identification of Possible Pathogenic Variants among Known Missence VUSs of Otoferlin

We used the best-created model (ConStruct, RF) and other methods mentioned in Table 1 with a balanced accuracy of over 0.8 to reveal the most possible pathogenic SAVs through a set of 1302 known variants of uncertain significance (VUSs) (see Material and Methods, Section 4.4.). The following requirements were used:

(1) The predicted value calculated using ConStruct (RF) for VUS was higher than 0.85.
(2) VUS was estimated as pathogenic by eight other methods based on the data from dbNSFP4.4 and SAV-Pred prediction.

As a result, 16 of the 1302 VUSs were selected as the most probable to be pathogenic (Table 2).

Table 2 presents the description of variants including their position in the protein sequence, reference and changing amino acid residues in this position, including the rs identifier of variants from the NCBI SNP database (dbSNP), the values of allele frequency from the GnomAD 3.1.1 database, the mean RankScore values calculated from the appropriate methods and dbNSFP4.4, and the value of prediction given by ConStruct (RF) and SAV-Pred methods. The variants are arranged in descending order by their mean RankScore values. The most probable pathogenic variants are at the top of the table. Only one variant (Asp1608Ala) had no record in dbSNP. Six variants had records in GnomAD with a very low frequency in the population. Five variants ranged from 456 to 562 positions, while others were from 1127 to 1687 positions.

## 3. Discussion

Assessing the impact of OTOF gene variants on the development of ANSD had an important scientific and applied significance as an increase in the accuracy of diagnosis of this disease can be widely used in clinical practice. The bioinformatics analysis of the currently available data using a machine learning algorithm can be used to establish patterns of the OTOF gene variants’ influence on the development of ANSD and, as a result, predict the risk of the disease based on the updated information on OTOF genetic variants.

The current work presents an approach to predicting the clinical consequences of genetic variants of OTOF based on an analysis of the structure and functions of the encoded otoferlin protein, as well as the intron–exon structure of the gene and conservativity features of nucleotide substitutions. Based on the OTOF gene variants data, for the clinically established consequences for hearing function, as well as a developed system of predictor variables, a classification model was built using the Random Forest machine learning algorithm. This created and validated model can foresee the risk of developing ANSD and has high predictive accuracy and reliability. When carrying out the 5-F CV procedure, the model demonstrates the stability of the results in all key metrics (accuracy, balanced accuracy, sensitivity, specificity, etc.). The model makes it possible to predict the risk of developing this disease based on targeted sequencing data with a balanced accuracy of more than 88% and, therefore, can be successfully used as an additional tool in solving problems of clinical and laboratory diagnostics in order to increase the accuracy and reliability of the molecular genetic diagnosis of ANSD. It was demonstrated that not all well-known methods work with a high pathogenicity prediction accuracy for the otoferlin missense variant. The list of the most accurate methods is given in Table 1.

Further improvement of the model may include the expansion of the training dataset considering the updated information on OTOF variants and optimizing the predictor system based on the latest molecular genetics and biochemical studies. The selection of the 16 most possible pathogenic missense variants is a demonstration of such an approach. Anyone can use the predicted results for 1302 VUSs to estimate other possible pathogenic variants (Supplements, Table S2). In addition, it is of interest to solve problems of classifying synonymous variants of the OTOF gene, which have a high level of uncertainty in terms of predicting the clinical phenotype.

The study displayed that there are relationships between the pathogenicity of SAVs and structural features of the gene/protein, and the score of the amino acid substitution based on BLOSUM also as nucleotide conservation in SNP positions. These characteristics are also recommended to be taken into account during the annotation of new OTOF SAVs.

Impaired speech perception is considered the leading clinical sign of ANSD despite the relative preservation of pure tone detection thresholds. The diagnosis of ANSD is complicated by limitations associated with the assessment of auditory perception at the prelingual stage of child development. The genetic testing of young children aimed at searching for mutations in the OTOF gene has an important diagnostic value due to the clear limitations in the use of subjective psychoacoustic methods (for example, speech or pure-tone audiometry). The results obtained can be used in the field of the molecular genetic testing of children with congenital hearing loss in order to increase the accuracy of diagnosis for auditory neuropathy spectrum disorder and timely implementation of the necessary therapeutic and rehabilitation measures in the early development of hearing pathology.

The approach used in this work to construct a model for the classification of genetic variants of OTOF based on the molecular structure analysis of the encoded protein, as well as the structure of the gene under study, can be extended to other currently known types of hearing pathology if the structure of the protein encoded by the gene under study is known.

## 4. Materials and Methods

### 4.1. Dataset for Modelling

The primary resource for data on OTOF gene variants was the Deafness Variation Database (DVD), curated by The Molecular Otolaryngology and Renal Research Laboratories, at the University of Iowa since 2011 [20]. DVD is a resource that collates and annotates all gene variants associated with hearing loss, as well as their clinical interpretation. The Deafness Variation Database provides a single classification for each variant based on the evidence collected and is curated by a multidisciplinary expert panel that includes clinicians, geneticists, and bioinformaticians.

The analysis of DVD data showed that single-nucleotide missense substitutions in the protein-coding (exon) regions of the OTOF gene are the most widely represented. These variants may be responsible for altering the ability of otoferlin to perform its function in the human auditory system. The identification of such genetic variants provides opportunities to predict the risk of ANSD in clinical practice. Not all missense variants lead to significant modifications of the protein, which is due to substitutions of amino acids with similar physicochemical properties. In addition, an amino acid substitution can affect areas of the protein that do not significantly affect its biological function. Difficulties in predicting the effect of missense variants on the risk of disease development are associated with the ambiguity of their effect on the protein product. This circumstance has led to the fact that a significant proportion of missense variants are variants of undetermined meaning (VUS, Variant of Uncertain Significance). When selecting cases for subsequent analysis, we relied on an empirical approach to determine the association of a genetic variant with an auditory neuropathy spectrum disorder based on epidemiological data. Thus, a missense variant (“missense_variant” or “missense_variant&splice_region_variant” labels should be included in the field “Consequence”) is considered to be harmful (pathogenic, likely pathogenic) or benign (benign, likely benign) based on known clinical evidence, i.e., a clinically observable phenotype. As a result, 264 variants were selected in the dataset. It is important to note that the DVD database does not use a single terminology to describe the clinical phenotype, as observations are integrated from various sources. Formulations were compared with the observations presented in other databases (Gene4HL, ClinVar), where the same phenotypes were represented as DFNB9 (OMIM: 603681). In addition, an analysis of recent studies allows us to conclude that the currently known hearing pathologies associated with mutations of the OTOF gene are ANSD (auditory neuropathy, autosomal recessive 1, and deafness, autosomal recessive 9). It is this phenotype that is the subject of interest in this study. The dataset was subsequently expanded to include additional observations from the Gene4HL: An Integrated Genetic Database for Hearing Loss (http://www.genemed.tech/gene4hl/home (accessed on 5 November 2023)) [21]. Most observations from the databases used were consistent. However, all unique (non-overlapping) observations were collected into a single dataset for the subsequent analysis. A special dataset of 270 missense variants was created as a result of this analysis (Supplements, Table S1).

### 4.2. Variables for Modelling

The dataset included the following information about missense variants and the affiliation of each observation to one of the two classes based on the assessment of the presence/absence of an individual carrier of ANSD (Class 1: Presence of ANSD, Class 2: Absence of ANSD). In order to predict the class, we developed a set of predictor variables based on modern ideas about the molecular structure of the OTOF gene, as well as the encoded otoferlin protein (long isoform) and conservativity features of nucleotide substitutions related to SAV:

1. *Position.* The position of SAV in the otoferlin sequence.
2. *Exon number*. The determination of the exon–intron structure of the gene showed that OTOF consists of 48 coding exons. Based on the data from the VEP service (Ensembl Variant Effect Predictor, https://www.ensembl.org/info/docs/tools/vep/index.html (accessed on 5 November 2023)) [22], the localization of genetic variants among 46 exons of the gene was established. For each observation in our dataset, we present a predictor variable, “exon number”, in which the genetic variant is located. This predictor is represented by values from 1 to 46.
3. *The significance of amino acid substitution.* The significance of substitutions in the otoferlin protein was assessed using the Blosum80 substitution matrix, which is used to align the sequences of closely related proteins. In accordance with the Blosum80 matrix, each amino acid substitution was assigned a score from 3 to 4. Thus, this predictor trait reflects the importance of amino acid substitution for the implementation of otoferlin functions in the functioning of the auditory analyzer.
4. *Functional domain.* According to molecular studies of the structure and functioning of otoferlin (InterPro database, https://www.ebi.ac.uk/interpro/ (accessed on 5 November 2023)) [23], in the long isoform of this protein, it is customary to distinguish six functional domains C2 and the C-terminal transmembrane (TM) domain. C2 domains are globular domains composed of antiparallel β-sheets that are known for their ability to bind to Ca^2+^ and phospholipids. The long isoform of the protein (1979 amino acid residues) is critical for normal hearing function. Of great importance for achieving this study’s goals was the analysis of genetic variants of the OTOF gene from the point of view of their influence on the functional domains of the encoded protein. The predictor value was assigned based on the data on the localization of amino acid substitutions in one of the functional domains or beyond them. The value of the predictor feature was the domain number (from 1 to 7) in which the change in amino acid change occurred or a of value 0 if the amino acid change was localized outside the functional domains.
  The positions of functional domains in otoferlin are as follows:
  − C2-1 (positions 1–98);
  − C2-2 (positions 236–357);
  − C2-3 (positions 400–531);
  − C2-4 (positions 944–1069);
  − C2-5 (positions 1115–1242);
  − C2-6 (positions 1464–1593);
  − C2-7 (positions 1714–1865).
5. *Region.* This structural element of the protein includes internally disordered regions (IDR). There are four regions in the tertiary structure of otoferlin. In accordance with the localization of the identified regions in the protein, each observation was assigned a value corresponding to the region number (1–4) or 0 if the amino acid substitution was outside these regions.
  Otoferlin regions:
  − 1 (positions 128–171);
  − 2 (positions 642–694);
  − 3 (positions 1299–1324);
  − 4 (positions 1343–1405).
6. *Composition-biased areas.* Such regions consist of a subset of amino acid residue types that are unevenly distributed along the length of the region. This predictor trait is based on the localization of the amino acid substitution in one of the established areas of low complexity. Observations were assigned a value of 1 if there was an amino acid substitution within one of the 5 defined low complexity regions and 0 if the substitution was outside these regions.
7. *Coiled-coil.* A coiled coil consists of alpha helices twisted together. Based on the data related to the localization of the structural element in question in the protein product, a predictor trait was generated that reflected the location of the amino acid substitution within the helical coil (value 1) or outside it (value 0).
8. *PhyloP100*. Evolutionary conservation values were calculated for nucleotide positions in relation to the appropriate SNP based on the multiple alignments of 100 vertebrate genomes. The data were extracted using the UCSC Table Browser from the appropriate track of the UCSC genome browser [24].
9. *PhyloP_30Primates.* Evolutionary conservation values were calculated for nucleotide positions related to the appropriate SNP based on the multiple alignments of 30 primate genomes. The data were also extracted using the UCSC Table Browser.

The data relating to regions, the coiled coil, and domains were based on UniProt information for the Q9HC10 record of human otoferlin, taking into account the 3D model of otoferlin made by AlphaFold.

The plots with the distribution of these characteristics according to the pathogenic and benign classification are provided in the Supplementary Material (Figures S13–S19). They showed that pathogenic variants have the following tendency: grouping in exons with a high number; a negative BLOSUM80 score for amino acid substitution; a more frequent appearance in 2–7 domains; localization between structured regions; and high values of evolutionary conservation PhyloP100 and PhyloP_30 primates.

### 4.3. Building and Validation of the Classification Models

The above-mentioned variables were calculated for each SAV, and the results were used as input for the creation of classification models using four well-known machine learning methods (Random Forest, Bayes, SVM, and Probabilistic Neural Network) in a freely available analytic platform KNIME (https://www.knime.com/ (accessed on 5 November 2023)).

The accuracy of this method was estimated by the five-fold cross-validation procedure (5-F CV) when each SAV was consequently annotated by the order of their position in the otoferlin sequence from 1 to 5. Then, five pairs of training and independent test sets were created based on this annotation. The characteristics of prediction accuracy were calculated using Scorer and ROC Curve nodes in KNIME.

### 4.4. Dataset of VUS Variants

The dataset of VUS was created based on the DVD database. It included 1302 VUS related to missense variants and ANSD. This dataset (see Supplements, Table S2) was used to determine potentially pathogenic SAVs with the best-created classification model and the most accurate known methods.

## Figures and Tables

**Table 1:** The prediction accuracy of the created method and other methods.

Method	TP	TN	FP	FN	Specificity	Sensitivity	BA ^1^	Accuracy	AUC
ConStruct, RF ^2^	76	162	15	17	0.817	0.915	0.866	0.881	0.903
ConStruct, Bayes ^2^	85	125	52	8	0.914	0.706	0.810	0.778	0.857
ConStruct, SVM ^2^	66	151	26	27	0.710	0.853	0.781	0.804	0.860
ConStruct, PNN ^2^	43	171	6	50	0.462	0.966	0.714	0.793	0.879
MetaLR ^3^ [10]	72	173	4	21	0.774	0.977	0.876	0.907	0.941
MetaSVM ^3^ [10]	71	172	5	22	0.763	0.972	0.868	0.900	0.947
MutationAssessor ^3^ [11]	77	161	16	16	0.828	0.910	0.869	0.881	0.932
PolyPhen 2 HDIV ^3^ [12]	82	155	22	11	0.882	0.876	0.879	0.878	0.921
PROVEAN ^3^ [13]	79	157	20	14	0.849	0.887	0.868	0.874	0.918
SIFT 4G ^3^ [14]	74	156	21	19	0.796	0.881	0.839	0.852	0.928
MutPred ^3^ [15]	54	113	12	18	0.75	0.904	0.827	0.848	0.887
LIST-S2 ^3^ [16]	88	122	55	5	0.946	0.689	0.818	0.778	0.922
SAV-Pred ^2^ [8]	76	139	38	17	0.817	0.785	0.801	0.796	0.876
FATHMM ^3^ [17]	46	170	7	47	0.495	0.960	0.728	0.800	0.684
M-CAP ^3^ [18]	89	76	82	3	0.967	0.481	0.724	0.660	0.911
MVP ^3^ [19]	91	64	105	2	0.978	0.379	0.679	0.592	0.922

^1^ BA—Balanced accuracy ((Specificity + Sensitivity)/2). ^2^ The values were given by 5-F CV. ^3^ The values were given based on dbNSFP4.4 data. TP—true positive, TN—true negative, FP—false positive, FN—false negative. AUC—Area under the receiver operating characteristic curve. The cutoffs provided by the authors of the methods were used to calculate accuracy based on dbNSFP4.4 data.

**Table 2:** The selected and most probable pathogenic variants related to ANSD through 1302 missense VUS.

Variant	rs_dbSNP151	Allele Frequency, GnomAD	Mean RankScore *
Leu1504Pro	rs775737412	-	0.925
Asp1608Tyr	rs111033425	0.0000263	0.919
Leu1537His	rs1171483566	-	0.911
Asp1608Ala	-	-	0.905
Gly1602Ser	rs776265576	-	0.904
Trp1606Gly	rs1218385801	-	0.892
Pro1128Leu	rs1465110068	-	0.889
Pro476Ser	rs573354216	0.0000066	0.873
Pro1628Thr	rs760176622	-	0.857
Gly562Ser	rs1415917697	0.0000131	0.849
Arg1127Gln	rs1408254984	0.0000132	0.846
Gly511Ser	rs1484959545	0.0000066	0.844
Glu1687Lys	rs1407487182	-	0.843
Val1498Gly	rs752201516	-	0.832
Ser542Phe	rs1467064287	-	0.822
Val456Met	rs753282692	0.0000131	0.799

* Mean RankScore—the mean value of RankScore values of the ten most accurate methods to predict pathogenic SAVs (Supplements, Table S3).

## Data Availability

All data on missense variants used in this study from DVD (https://deafnessvariationdatabase.org/, (accessed on 14 September 2023)), Gene4HL (http://www.genemed.tech/gene4hl/home, (accessed on 14 September 2023)), gnomAD (http://gnomad-sg.org/, (accessed on 12 October 2023)) and dbNSFP4.4 (http://database.liulab.science/dbNSFP, (accessed on 14 September 2023)) databases are provided in Excel files (Tables S1–S3) and Figures S1–S19 are provided in the PDF file (Supplements.pdf) in the Supplementary Materials.

## Supplementary Materials

The following supporting information can be downloaded at: https://www.mdpi.com/article/10.3390/ijms242417240/s1.
