# Supplementary material for: Predicting the Impact of OTOF Gene Missense Variants on Auditory Neuropathy Spectrum Disorder
Source: Int J Mol Sci. 2023 Dec 7;24(24):17240. doi: 10.3390/ijms242417240 (PMC10743402; doi:10.3390/ijms242417240)
Supplement: Supplementary file 1 [file ijms-24-17240-s001.zip › Supplements.pdf]

Supplements

to the paper of Dmitry A. Dmitriev, Boris V. Shilov, Michail M. Polunin, Anton D. Zadorozhny and Alexey A. Lagunin

"Predicting the impact of OTOF gene missense variants on auditory neuropathy spectrum disorder"

Contents

Figure S1. The distribution of MetaLR prediction results for benign and pathogenic variants. 2

Figure S2. The distribution of MetaSVM prediction results for benign and pathogenic variants. 3

Figure S3. The distribution of MutationAssessor prediction results for benign and pathogenic variants. 4

Figure S4. The distribution of PolyPhen 2 HDIV prediction results for benign and pathogenic variants. 5

Figure S5. The distribution of PROVEAN prediction results for benign and pathogenic variants. 6

Figure S6. The distribution of SIFT 4G prediction results for benign and pathogenic variants. 7

Figure S7. The distribution of MutPred prediction results for benign and pathogenic variants. 8

Figure S8. The distribution of LIST-S2 prediction results for benign and pathogenic variants. 9

Figure S9. The distribution of SAV-Pred prediction results for benign and pathogenic variants. 10

Figure S10. The distribution of FATHMM prediction results for benign and pathogenic variants. 11

Figure S11. The distribution of M-CAP prediction results for benign and pathogenic variants. 12

Figure S12. The distribution of MVP prediction results for benign and pathogenic variants. 13

Figure S13. The distribution of exon numbers for benign and pathogenic variants. 14

Figure S14. The distribution of amino acid substitution BLOSUM80 scores for benign and pathogenic variants. 15

Figure S15. The distribution of functional domain values for benign and pathogenic variants. 16

Figure S16. The distribution of composition-biased area values for benign and pathogenic variants. 17

Figure S17. The distribution of coiled-coil values for benign and pathogenic variants. 18

Figure S18. The distribution of PhyloP100 values for benign and pathogenic variants. 19

Figure S19. The distribution of PhyloP_30Primates values for benign and pathogenic variants. 20

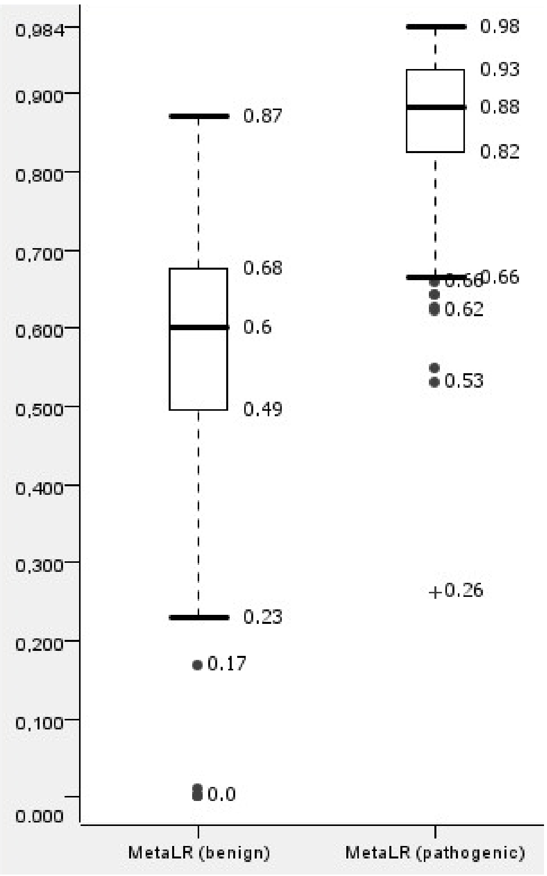

Figure S1. The distribution of MetaLR prediction results for benign and pathogenic variants. "+" means extreme outliers; ● means mild outliers.

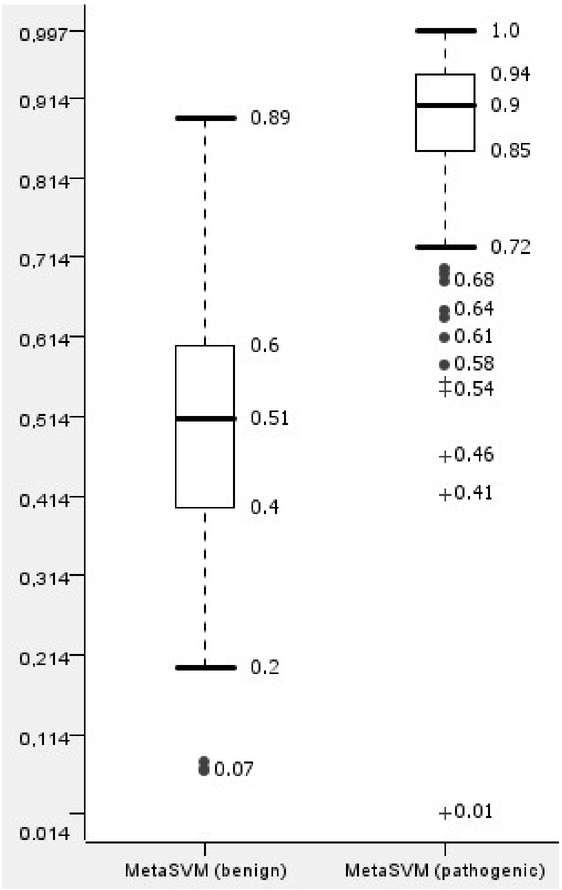

Figure S2. The distribution of MetaSVM prediction results for benign and pathogenic variants. "+" means extreme outliers; ● means mild outliers.

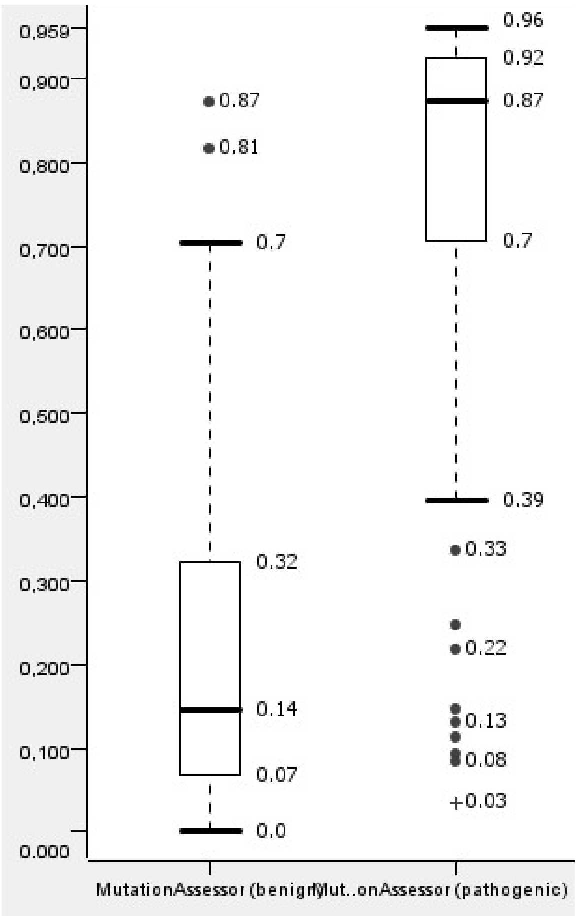

Figure S3. The distribution of MutationAssessor prediction results for benign and pathogenic variants. "+" means extreme outliers; ● means mild outliers.

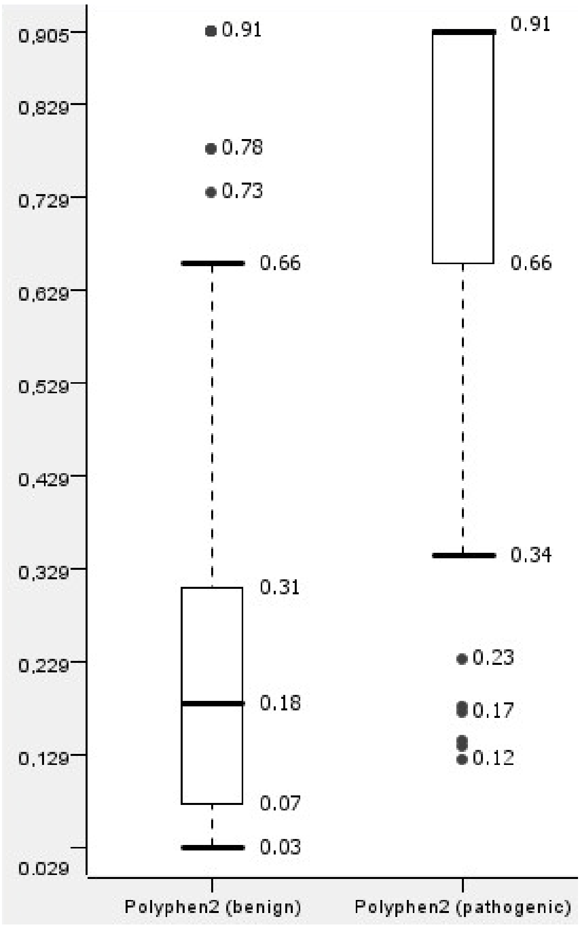

Figure S4. The distribution of PolyPhen 2 HDIV prediction results for benign and pathogenic variants. ● means mild outliers.

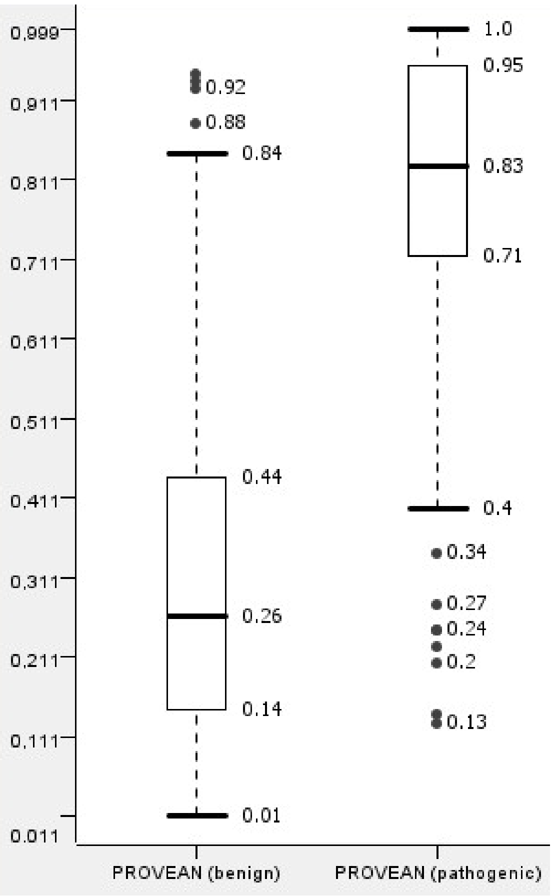

Figure S5. The distribution of PROVEAN prediction results for benign and pathogenic variants. ● means mild outliers.

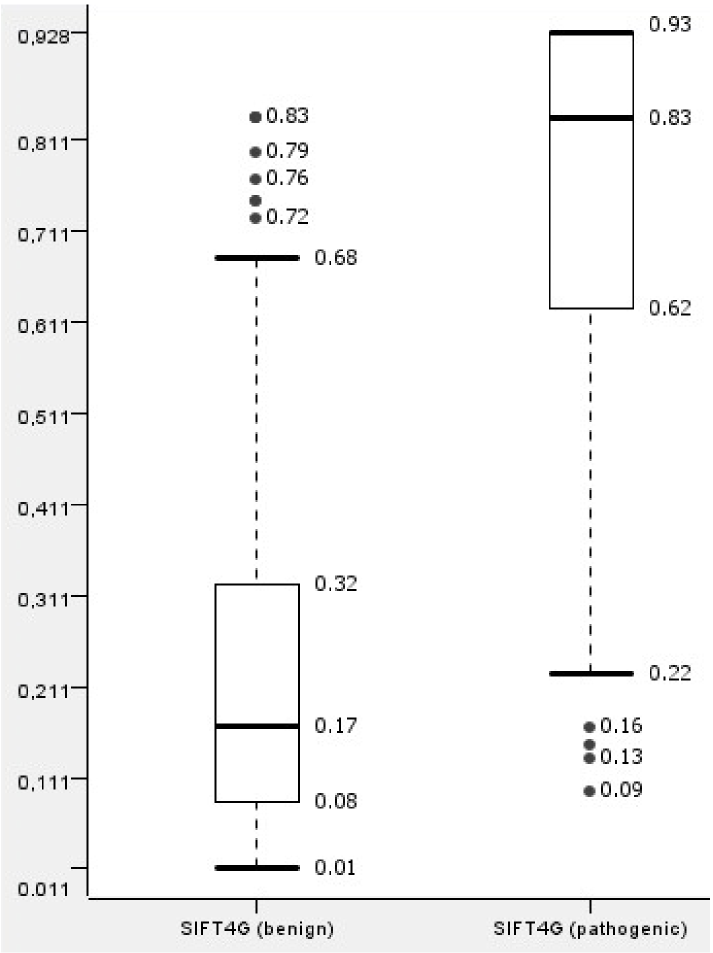

Figure S6. The distribution of SIFT 4G prediction results for benign and pathogenic variants. ● means mild outliers.

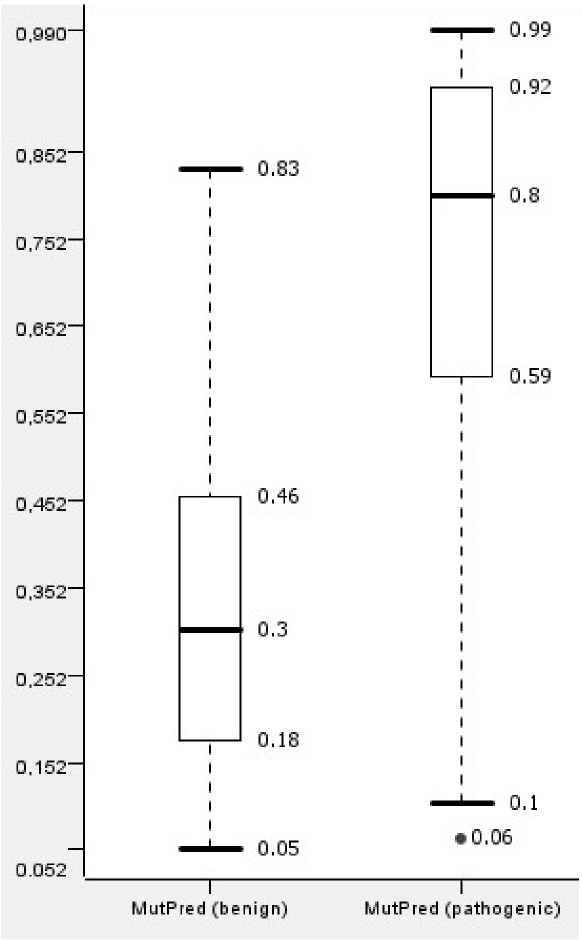

Figure S7. The distribution of MutPred prediction results for benign and pathogenic variants. ● means mild outliers.

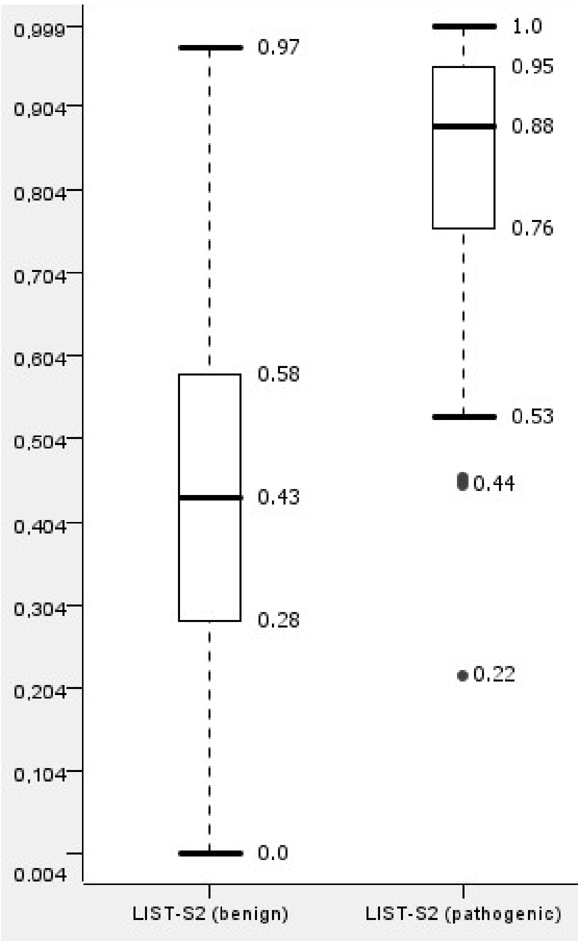

Figure S8. The distribution of LIST-S2 prediction results for benign and pathogenic variants. ● means mild outliers.

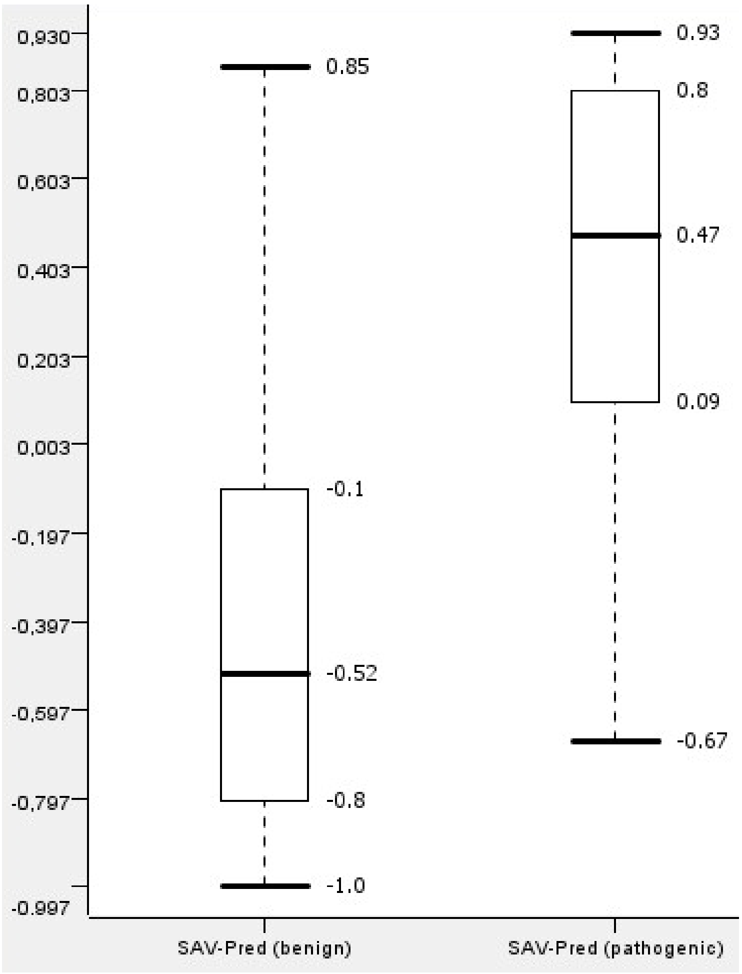

Figure S9. The distribution of SAV-Pred prediction results for benign and pathogenic variants.

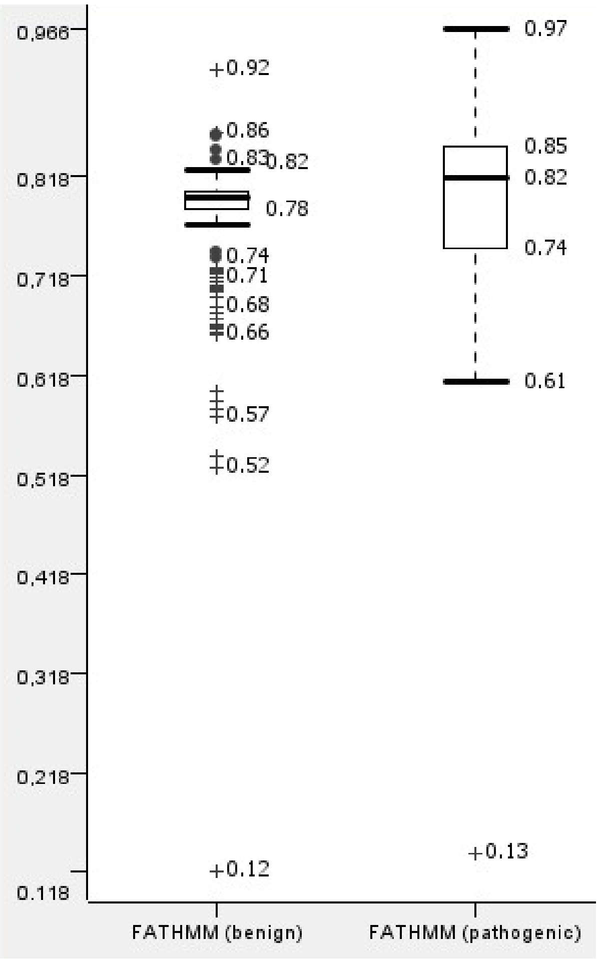

Figure S10. The distribution of FATHMM prediction results for benign and pathogenic variants. "+" means extreme outliers; ● means mild outliers.

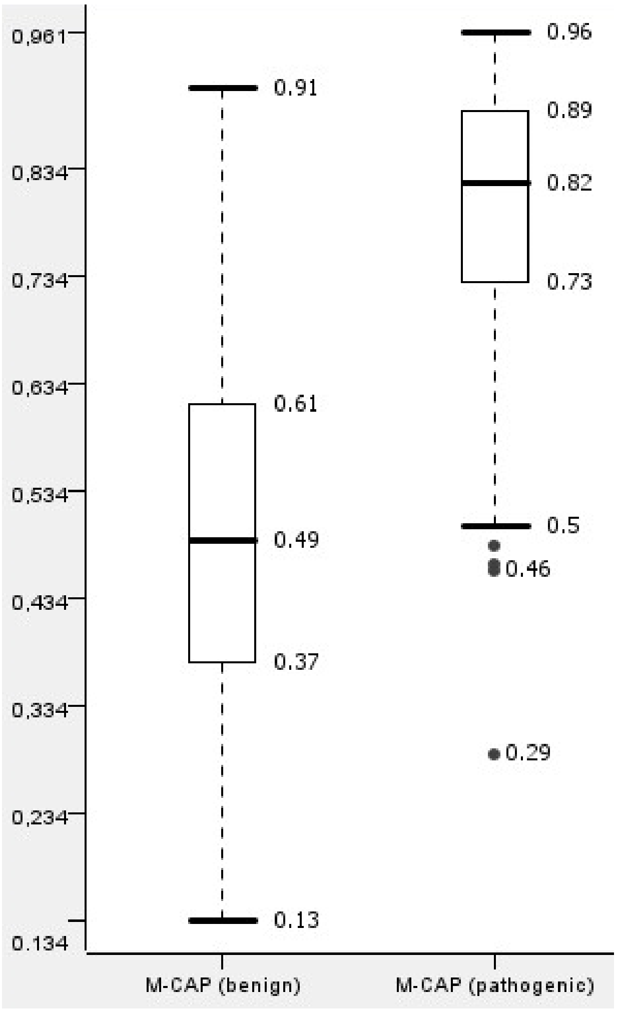

Figure S11. The distribution of M-CAP prediction results for benign and pathogenic variants. ● means mild outliers.

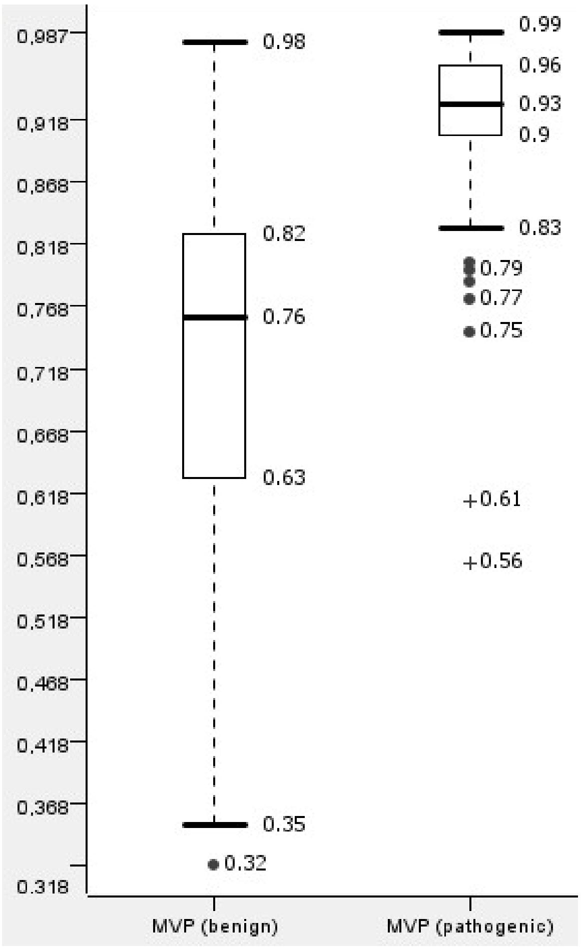

Figure S12. The distribution of MVP prediction results for benign and pathogenic variants. "+" means extreme outliers; ● means mild outliers.

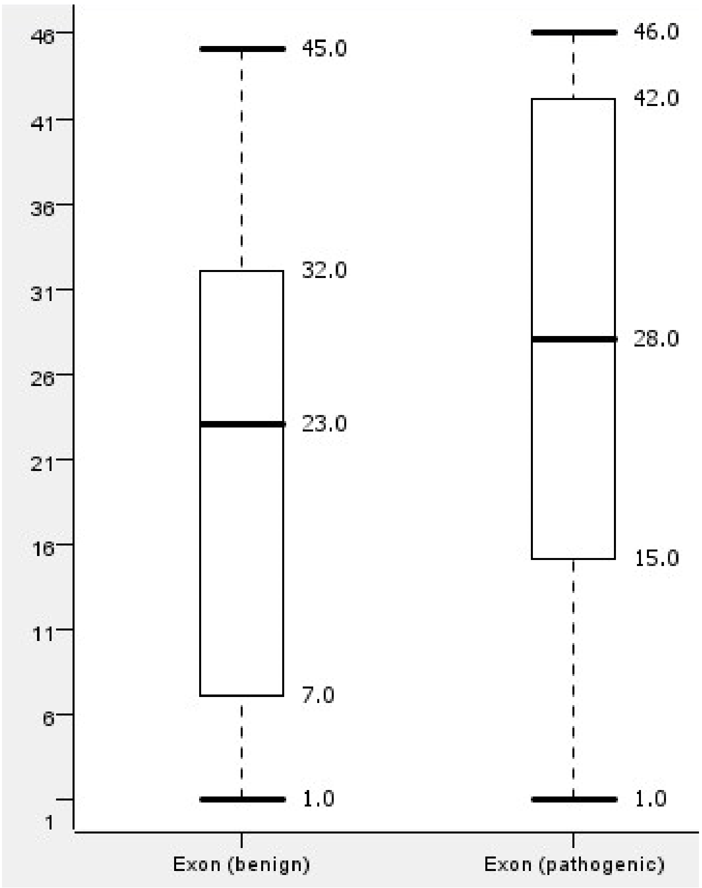

Figure S13. The distribution of exon numbers for benign and pathogenic variants.

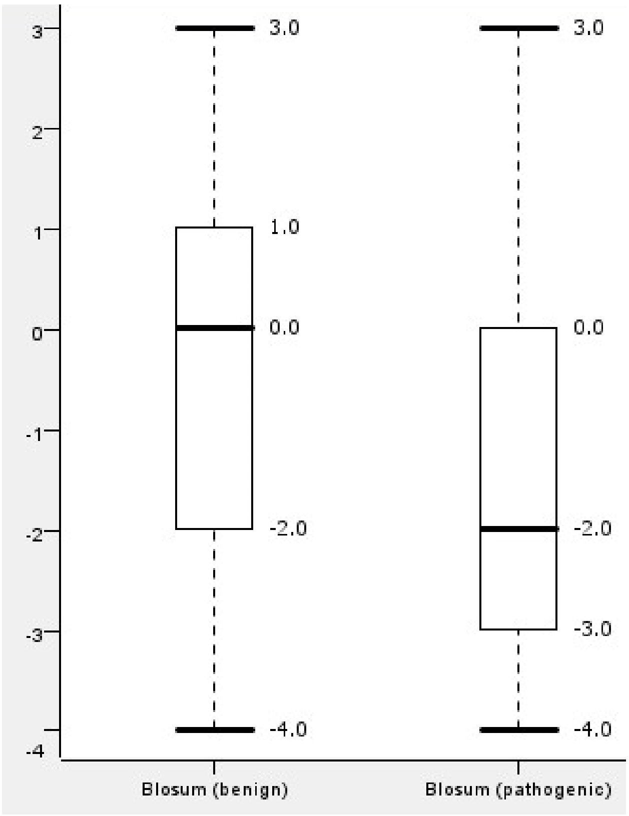

Figure S14. The distribution of the significance of amino acid substitution BLOSUM80 scores for benign and pathogenic variants.

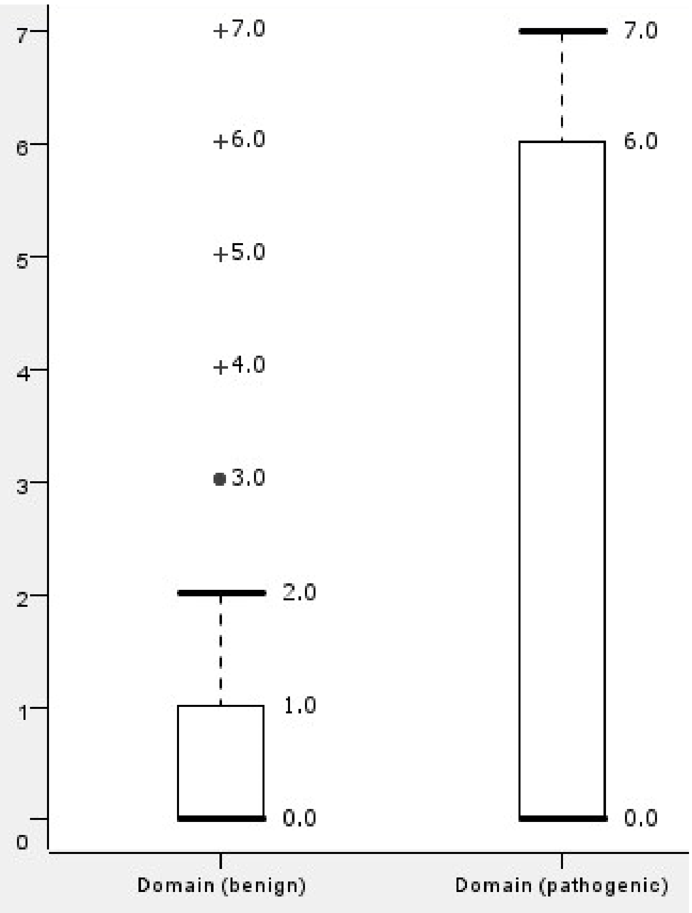

Figure S15. The distribution of functional domain values for benign and pathogenic variants. “+” means extreme outliers; ● means mild outliers.

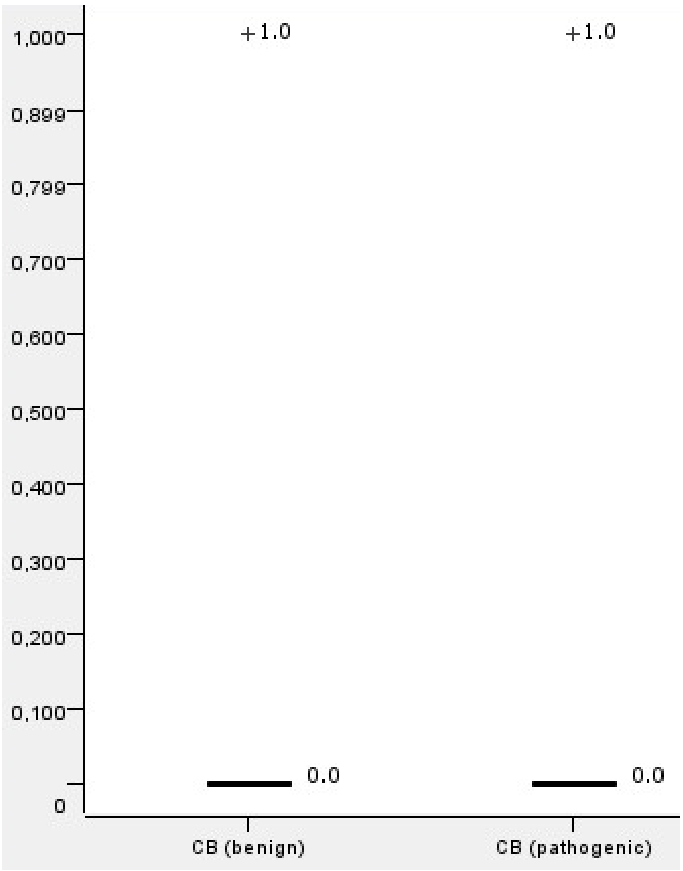

Figure S16. The distribution of composition-biased (CB) area values for benign and pathogenic variants. "+" means extreme outliers.

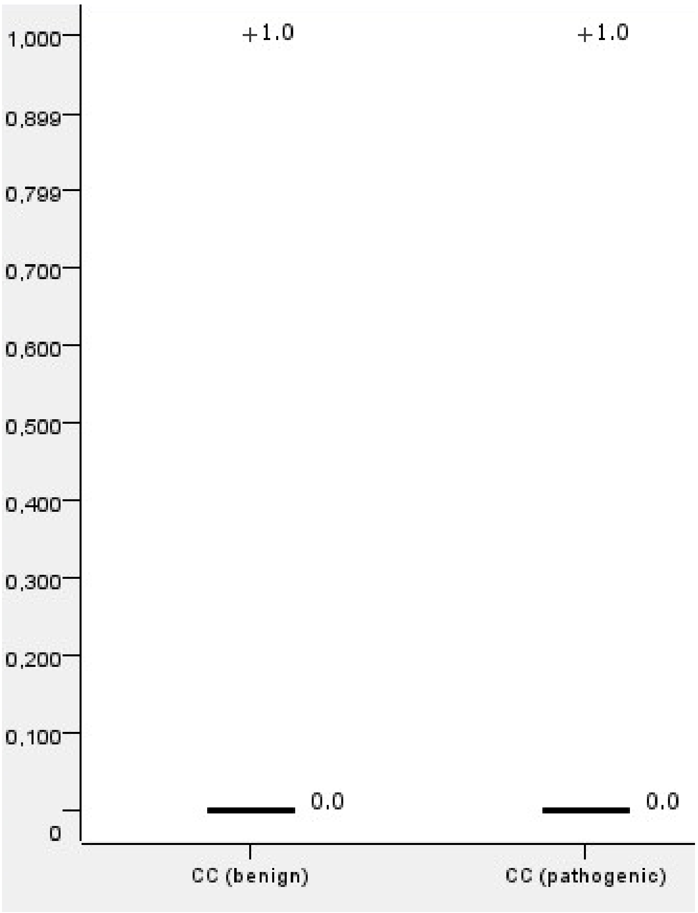

Figure S17. The distribution of coiled-coil (CC) values for benign and pathogenic variants. “+” means extreme outliers.

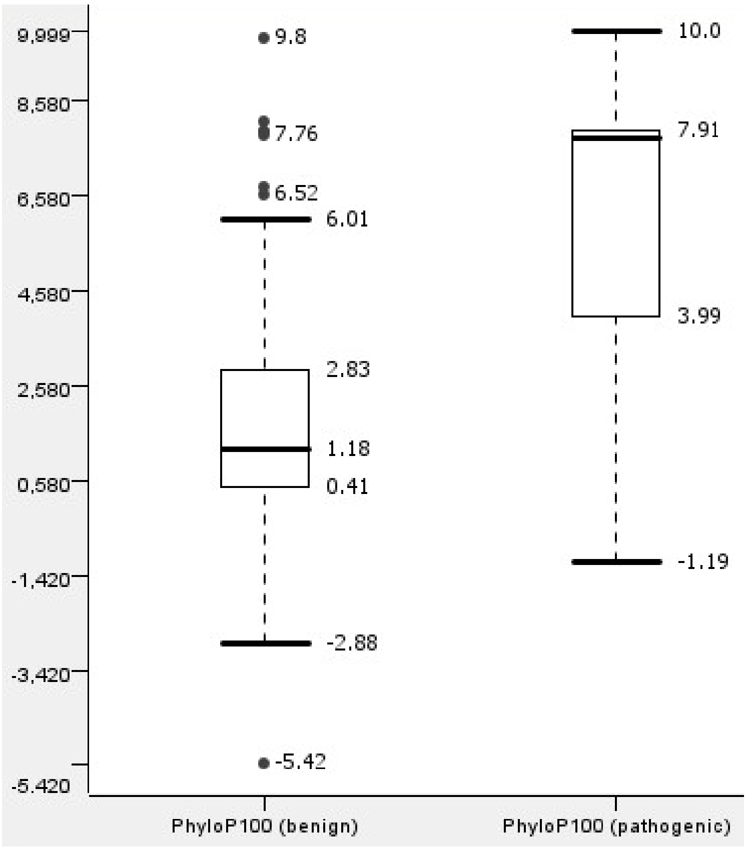

Figure S18. The distribution of PhyloP100 values for benign and pathogenic variants. ● means mild outliers.

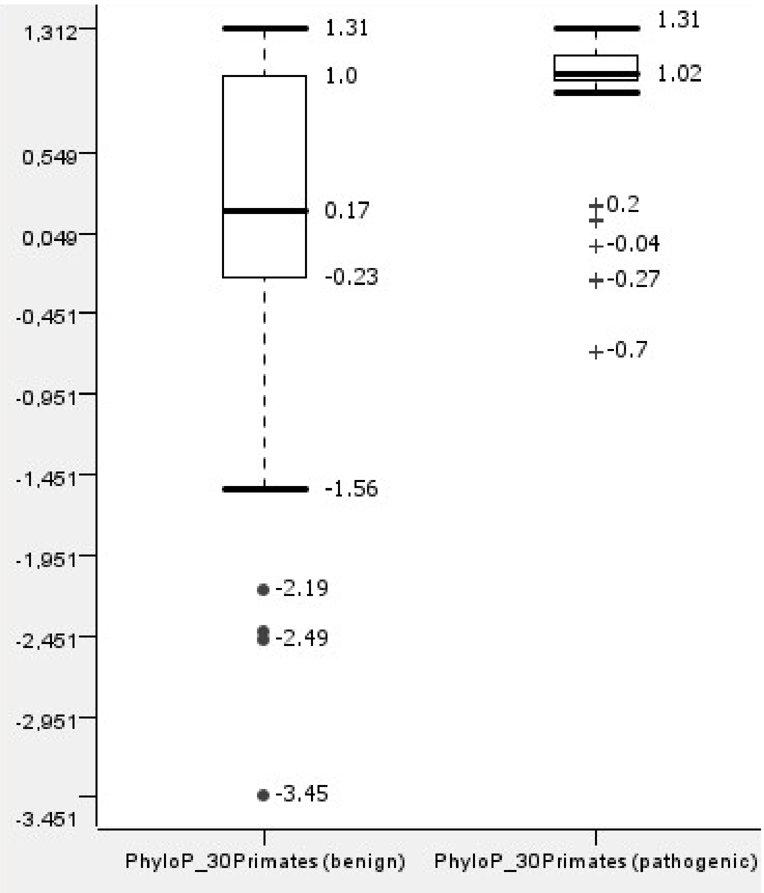

Figure S19. The distribution of PhyloP_30Primates values for benign and pathogenic variants. "+" means extreme outliers; ● means mild outliers.
